# Coronavirus Disease (COVID-19): Comprehensive Review of Clinical Presentation

**DOI:** 10.3389/fpubh.2020.582932

**Published:** 2021-01-15

**Authors:** Om Prakash Mehta, Parshal Bhandari, Akshay Raut, Salah Eddine Oussama Kacimi, Nguyen Tien Huy

**Affiliations:** ^1^Department of Medicine, King Edward Medical University/ Mayo Hospital, Lahore, Pakistan; ^2^Department of Anesthesia and Intensive Care, Post-Graduate Medical Institute/LGH, Lahore, Pakistan; ^3^Rajarshee Chhatrapati Shahu Maharaj Government Medical College, Kolhapur, India; ^4^Department of Medicine, Faculty of Medicine, University of Tlemcen, Tlemcen, Algeria; ^5^School of Tropical Medicine and Global Health, Nagasaki University, Nagasaki, Japan; ^6^Institute of Research and Development, Duy Tan University, Da Nang, Vietnam

**Keywords:** SARS-CoV-2, Covid-19, symptomatology, clinical presentation, signs and symptoms, clinical features, coronavirus

## Abstract

COVID-19 is a rapidly growing pandemic with its first case identified during December 2019 in Wuhan, Hubei Province, China. Due to the rampant rise in the number of cases in China and globally, WHO declared COVID-19 as a pandemic on 11th March 2020. The disease is transmitted via respiratory droplets of infected patients during coughing or sneezing and affects primarily the lung parenchyma. The spectrum of clinical manifestations can be seen in COVID-19 patients ranging from asymptomatic infections to severe disease resulting in mortality. Although respiratory involvement is most common in COVID-19 patients, the virus can affect other organ systems as well. The systemic inflammation induced by the disease along with multisystem expression of Angiotensin Converting Enzyme 2 (ACE2), a receptor which allows viral entry into cells, explains the manifestation of extra-pulmonary symptoms affecting the gastrointestinal, cardiovascular, hematological, renal, musculoskeletal, and endocrine system. Here, we have reviewed the extensive literature available on COVID-19 about various clinical presentations based on the organ system involved as well as clinical presentation in specific population including children, pregnant women, and immunocompromised patients. We have also briefly discussed about the Multisystemic Inflammatory Syndrome occurring in children and adults with COVID-19. Understanding the various clinical presentations can help clinicians diagnose COVID-19 in an early stage and ensure appropriate measures to be undertaken in order to prevent further spread of the disease.

## Introduction

COVID-19 is a growing pandemic with initial cases identified in Wuhan, Hubei province, China toward the end of December 2019. Labeled as Novel Coronavirus 2019 (2019-nCoV) initially by the Chinese Center for Disease Control and Prevention (CDC) which was subsequently renamed as Severe Acute Respiratory Syndrome-Coronavirus-2 (SARS-CoV-2) due to its homology with SARS-CoV by the International Committee on Taxonomy of Viruses (ICTV) (1, 2). The World Health Organization (WHO) later renamed the disease caused by SARS-CoV-2 as Coronavirus Disease-2019 (COVID-19) (3). COVID-19 is primarily a disease of the respiratory system affecting lung parenchyma with fever, cough, and shortness of breath as the predominant symptoms. Recent studies have shown that it can affect multiple organ systems and cause development of extra-pulmonary symptoms. Presence of extra-pulmonary symptoms can often lead to late diagnosis and sometimes even mis-diagnosis of COVID-19 which can be detrimental to patients. As researchers globally continue to understand COVID-19 and its implications on the human body, knowledge about the various clinical presentations of COVID-19 is paramount in early diagnosing and treatment in order to decrease the morbidity and mortality caused by the disease.

## Epidemiology and Pathophysiology

While studying the early transmission dynamics of COVID-19 outbreak in Wuhan, many cases were found to be linked to the Huanan wholesale seafood market. Further investigation revealed <10% of the total cases could be linked to the market which led to the conclusion of human-to-human transmission of the virus occurring through respiratory droplets and contact transmission contributing to the rise in the number of affected individuals (4). The exponential rise in the number of cases in China and reporting of cases outside China in multiple countries led WHO to declare COVID-19 as a pandemic on 11th March 2020 (5).

SARS-CoV-2 tends to infect all age groups and is transmitted via direct contact or respiratory droplets generated during coughing or sneezing by the infected patient during both symptomatic or pre-symptomatic phase of infection. Other routes of transmission include fecal-oral route and fomites along with small risk of vertical transmission from mother to child if infection occurs during third trimester of pregnancy (6, 7). There has also been evidence of asymptomatic transmission of COVID-19 (8). The concept of super spreaders in relation to COVID-19 is emerging where a single individual either symptomatic or asymptomatic can infect a disproportionately large number of individuals in an appropriate super spreading conditions such as mass gathering due to production of large number of infectious agent for prolonged duration of time (9). As per the literature, the incubation period of COVID-19 ranges from 2 to 14 days with a mean incubation period of 3 days (10). The basic Reproduction number (Ro) of SARS-CoV-2 is 2–2.5. Each individual infected with COVID-19 can infect 2–2.5 other individuals in a naïve population which also explains the exponential growth in the number of cases (10). The disease tends to be of mild to moderate severity in roughly 80% of patients, and severe disease is associated with infants, elderly patients above 65 years, and patients with other comorbidities such as diabetes mellitus, hypertension, coronary artery disease, and other chronic conditions (1, 2). COVID-19 has also been found to be more severe in males than in females with a case fatality rate of 2.8% in males and 1.7% in females (11). The major organ system affected by the virus is the respiratory system, but it can affect other organ systems either directly or by the effect of host immune response. SARS-CoV-2, the causative agent of COVID-19, after entering the human host initially replicates in the epithelial mucosa of the upper respiratory tract (nose and pharynx) followed by migration to the lungs where further replication of virus occurs causing transient viraemia. The virus uses Angiotensin Converting Enzyme 2 (ACE2) receptor as a primary entry to cells. ACE2 is found abundantly in the mucosal lining of the respiratory tract, vascular endothelial cells, heart, intestine, and kidney. Thus, the virus has potential for replication in all these organs. After entry into cells, the virus undergoes further rapid replication within the target cells and induces extensive epithelial and endothelial dysfunction leading to exponential inflammatory response with the production of a large amount of proinflammatory cytokines and chemokines. Activation of proinflammatory cytokines and chemokines leads to neutrophil activation and migrations and results in the characteristic cytokine storm. The immunological downregulation of ACE2 by the virus contributes to acute lung injury in COVID-19. ACE2 also regulates the renin angiotensin system (RAS); thus, downregulation of ACE2 also causes dysfunction of RAS which contributes to enhanced inflammation (2, 11–15). These entire factors contribute to symptoms of COVID-19 with sepsis, multi-organ dysfunction, acute respiratory distress syndrome (ARDS), and prothrombotic state leading to an exacerbation of organ dysfunction.

## Clinical Manifestation

We review here the system based clinical features of COVID-19.

### Respiratory

According to report from WHO-China-Joint Mission on COVID-19, 55,924 laboratories confirmed cases of COVID-19 had fever (87.9%), dry cough (67.7%), fatigue (38.1%), sputum production (33.4%), difficulty breathing (18.6%), sore throat (13.9%), chills (11.4%), nasal congestion (4.8%), and hemoptysis (0.9%) (1).

Some patients may rapidly progress to acute lung injury and ARDS with septic shock. The median interval between the onset of initial symptoms to development of dyspnea, hospital admission, and ARDS was 5, 7, and 8 days respectively (10). Some patients with COVID-19 may have reduced oxygen saturation in blood (≤ 93%) with oxygen saturation down to 50 or 60% but remained stable without significant distress, and as such, were termed as salient hypoxia or happy hypoxia (16, 17). Trial of oxygen therapy, prone positioning, high flow continuous positive airway pressure, non-re-breathable mask alongside trial of anticoagulation are often used to manage these patients (16, 17). However, further study is required to define the role of these strategies in management.

The most frequent radiological abnormality among 975 patients with COVID-19 in computed tomography (CT) scan of chest was ground glass opacity (56.4%) and bilateral patchy shadowing (51.8%) (18). A scientific review of 2,814 patients have shown that the most common chest CT finding in COVID-19 patients was ground glass opacity followed by consolidation. However, the findings can vary in different patients and at various stages of diseases. Other CT findings include interlobular septal thickening, reticular pattern, crazy paving, etc. Atypical findings like air bronchogram, bronchial wall thickening, nodule, pleural effusion, and lymphadenopathy have also been noted in some studies (19). A study showed that among 877 patients with non-severe diseases and 173 patients with severe diseases, 17.9 and 2.9% of the patients did not have any detectable radiological abnormalities, respectively (18).

### ENT (Ear, Nose, and Throat)

ENT manifestations are one of the most frequent symptoms encountered by physicians in COVID-19. A peculiar clinical presentation in some COVID-19 patients includes the deterioration of sense, taste (dysgeusia), and loss of smell (anosmia). A systematic review and meta-analysis of 10 studies with 1,627 participants surveyed for olfactory deterioration and 9 studies with 1,390 participants examined for gustatory symptoms demonstrated prevalence of 52.73 and 43.93% of these symptoms among COVID-19 patients, respectively. These clinical features may often present at earlier stages of the disease (20). Additionally, sore throat, rhinorrhea, nasal congestion, tonsil edema, and enlarged cervical lymph nodes are commonly seen among otolaryngological dysfunctions in patients (21). A large observational study of 1,099 COVID-19 patients reported tonsils swelling in 23 patients (2.1%), throat congestion in 19 patients (1.7%) and enlarged lymph nodes in 2 patients (0.2%) (18). This can be explained by the fact that there is a high expression of ACE2 receptors on the epithelial cells of the oral and nasal mucosa including the tongue. It has been known that the novel coronavirus has a strong binding affinity to ACE2 receptors through which it invades host cells (22). This theory may explain the exhibition of extra-respiratory symptoms including ENT manifestations as part of COVID-19 symptoms.

### Cardiovascular

Cardiac manifestation in patient with COVID-19 can occur due to cardiac strain secondary to hypoxia and respiratory failure, direct effect of SARS-CoV-2 on heart or secondary to inflammation and cytokine storm, metabolic derangements, rupture of plaque and coronary occlusion by thrombus, and consequences of drugs used for treatment (23–25). The need for intensive care admission, non-invasive ventilation (46.3 vs. 3.9%), and invasive mechanical ventilation (22 vs. 4.2%) were higher among patients with cardiac ailments as compared to those without cardiac involvement as well as higher hospital mortality than those without myocardial involvement (51.2 vs. 4.5%) (26). These patients tend to have electrocardiographic (ECG) changes as well as elevations in high sensitivity cardiac troponin (hsCTn) and N- terminal pro-B-type natriuretic peptide (NT proBNP) which corresponded to raised inflammatory markers. Hypertension, acute and fulminant myocarditis, ventricular arrhythmias, atrial fibrillation, stress cardiomyopathy, hypotension and heart failure, acute coronary syndrome (ACS) with ST elevation or depression MI with normal coronaries have been reported (23, 27). In a Chinese cohort of 138 patients, 16.7% had arrhythmias with risk higher among those needing ICU care with no mention of the type of arrhythmia that was present (28). Less frequently, cardiac symptoms like chest pain or tightness and palpitation can be the initial presenting features without fever producing a diagnostic dilemma. Some of these patients eventually go on to develop respiratory symptoms as diseases progress (29). Patients who have recovered from acute illness may develop arrhythmias as a result of myocardial scar and need future monitoring (27). One important point to note is use of Renin Angiotensin Aldosterone System (RAAS) modulators in patients with COVID-19. Guidelines from ACC/AHA/HFSA recommends continuing them in high risk patient based on goal directed therapy approach supported by a recent systematic review and meta-analysis conducted by Hasan et. Al. which demonstrated use of ACEI/ARB in COVID-19 patients is associated with lower odds/ hazards of mortality and development of severe/critical diseases as compared to no use of ACEI/ARB (30, 31).

### Gastrointestinal

In the initial cohort of patients from China, nausea or vomiting and diarrhea were present in 5 and 3.7% of patients (1). Review of data from 2,023 patients showed anorexia to be the most frequently occurring gastrointestinal symptom in adults. Diarrhea was the most common presenting gastrointestinal symptom in both adults and children while vomiting was found to be more common in children (32). Other rare symptoms included nausea, abdominal pain, and gastrointestinal bleeding. There have been few instances where COVID-19 patients presented with only gastrointestinal symptoms without the development of fever or respiratory symptoms at the onset and during disease progression (33). In a smaller cohort of 204 patients, 50.5% had some form of intestinal symptoms and of those, 5.8% had only intestinal symptoms while the remaining patients developed respiratory symptoms subsequently. The most common symptoms reported among them was anorexia (78.64%), non-dehydrating diarrhea (34%), vomiting (3.9%), and abdominal pain (1.94%) (34). In addition, those with GI symptoms tend to have a longer interval between symptom onset and hospital admission (9 vs. 7.3 days) possibly due to lack of clinical suspicion and delay in diagnosis. Patients with gastrointestinal symptoms tend to have higher elevation in AST and ALT indicating coexistent liver injury (34). The mechanism behind GI illness is not clearly known but could be due to direct invasion of virus via ACE2 receptor in the intestinal mucosa. This can be supported by the fact that viral RNA can be detected in stool samples of COVID-19 patients which may also hint toward possible fecal-oral transmission (35). Liver dysfunction is likely secondary to the use of hepatotoxic drugs, hypoxia induced liver injury, systemic inflammation, and multi organ failure (36).

### Renal

Renal manifestation in patients with COVID-19 can occur due to direct invasion of podocytes and proximal tubular cells by SARS-CoV-2 virus, secondary endothelial dysfunction causing effacement of foot process with vacuolation and detachment of podocytes, and acute proximal tubular dysfunction (37). Furthermore, hypoxia, cytokine storm, rhabdomyolysis, nephrotoxic drugs, and overlying infections can all exacerbate renal injury (38). Based on initial reports, prevalence of Acute Kidney Injury (AKI) among COVID-19 hospitalized patients range from 0.5 to 29%. In a cohort of 701 patients, proteinuria (43.9%), hematuria (26.7%), elevated creatinine (14.4%), elevated blood urea nitrogen (13.1%), and low glomerular filtration rate (≤ 60 ml/min/1.73 m^2^) (13.1%) were present at the time of hospital admission with 5.1% developing AKI during the illness. AKI was more prevalent among those with baseline renal impairment (39). In another large cohort of 5,449 patients, 36.6% had AKI with prevalence higher among mechanically ventilated patients compared to non-ventilated patients (89.7 vs. 21.7%) (40). Patients developing renal impairment are prone to have higher mortality within the hospital. Another point to highlight is the presentation of COVID-19 in renal transplant recipients. Due to immunosuppression, these patients are likely to have low fever at presentation with swift clinical decline and requirement for mechanical ventilation with high mortality as compared to the general population (41).

### Neurological

Most patients with COVID-19 develop neurological symptoms along with respiratory symptoms during the course of illness; however, several case reports in review of literature document patient presentation of neurological dysfunction without typical symptoms of fever, cough, and difficulty breathing (42). There is a 2.5-fold enhanced risk of severe illness and increased death in patients with a history of previous stroke with similar findings among those with Parkinson's diseases. The prevalence of neurological features ranges from 6 to 36% along with hypoxic ischemic encephalopathy up to 20% in some series of patients (43). Neurological symptoms tend to occur early in the course of illness (median 1–2 days) with most common neurological features being headache, confusion, delirium, anosmia or hyposmia, dysgeusia or ageusia, altered mental status, ataxia, and seizures (44). Among patients admitted with COVID-19, the prevalence of ischemic stroke ranges from 2.5 to 5% despite receiving prophylaxis for venous thromboembolism. Patients prone to have established cardiovascular risk factors are likely to have a more severe diseases (43). Other presentations include viral encephalitis, acute necrotizing encephalopathy (ANE), infectious toxic encephalopathy, meningitis, Guillain Barre Syndrome (GBS), Miller Fisher syndrome, and polyneuritis cranialis with GBS being the first feature of COVID-19 in few cases (42, 43, 45). In COVID-19 patients, CNS features are possibly due to direct invasion of neurons and glial cells by SARS-CoV-2 as well as by endothelial dysfunction of blood brain barrier (BBB). Virus can gain access to CNS via hematogenous spread or retrograde movement across the olfactory bulb. The virus can be detected in CSF by RT-PCR and on brain parenchyma during autopsy. The fact that most patients develop anosmia or hyposmia during illness support this theory (45). After entry, the virus can cause reactive gliosis with activation of the inflammatory cascade. The combination of systemic inflammation, cytokine storm, and coagulation dysfunction can impair BBB function and alter brain equilibrium causing neuronal death (42).

### Ocular

Ocular manifestations can vary from conjunctival injection to frank conjunctivitis. In a Chinese cohort of 38 patients, 31.6% had ocular symptoms consisting primarily of conjunctivitis while conjunctival hyperemia, foreign body sensation in eye, chemosis, tearing or epiphora were more common among severe COVID-19 patients. Among them SARS-CoV-2 can be demonstrated in conjunctival as well as nasopharyngeal swab in 5.2% of patients, indicating a potential route for viral transmission (46). Conjunctivitis or tearing can be the initial presenting symptoms of COVID-19. Despite this fact, there is no documented case of severe ocular features relating to COVID-19.

### Cutaneous

Similar to other viral infections, SARS-CoV-2 can also produce varied dermatological features. A study of 88 patients from Italy showed that about 20.4% had some form of skin manifestations with 44.4% developing features at onset and duration of the disease progression (47). Maculopapular exanthem (36.1%) was identified as most common dermatological features followed by papulovesicular rash (34.7%), painful acral red purple papules (15.3%), urticaria (9.7%), livedo reticularis (2.8%), and petechiae (1.4%) (48). A study of 375 COVID-19 cases in Spain identified five different patterns of cutaneous manifestations in patients: acral areas of erythema with vesicles or pustules (pseudo-chilblain) (19%), other vesicular eruptions (9%), urticarial lesions (19%), maculopapular eruptions (47%), and livedo or necrosis (6%) (49). Majority of patients had lesions on the trunk with some experiencing lesions on hands and feet. There are case reports of COVID-19 associated with erythema multiforme and Kawasaki Disease in children (50, 51). Pathogenesis behind skin involvement remains unclear with some features explained by small vessel vasculitis, thrombotic events like DIC, hyaline thrombus formation, acral ischemia, or the direct effect of the virus like other viral illnesses (52).

### Musculoskeletal

The initial report from China revealed 14.8% of patients had myalgia or arthralgia among 55,924 COVID-19 patients. A review article reports that of 12,046 patients, fatigue was identified in 25.6% and myalgia and/or arthralgia in 15.5% with most patients reporting symptoms from the start of illness (53). There are reports suggesting myositis and rhabdomyolysis with markedly elevated creatinine kinase can occur during COVID-19 illness especially in patients with severe diseases and multi organ failure. Additionally, in some patients, rhabdomyolysis has been documented as the initial presentation of COVID-19 illness without typical respiratory symptoms (54, 55). A case series of four patients developing acute arthritis during hospital admission for COVID-19 has been reported with exacerbation of crystal arthropathy (gout and calcium pyrophosphate diseases) but negative for SARS-CoV-2 RT-PCR in synovial fluid (56). Treatment with steroids and colchicine was used in all four cases. An important consideration to note was that all four patients developed arthritis despite previous treatment with immunomodulatory therapy (hydroxychloroquine, tocilizumab, and pulse methylprednisolone).

### Hematological

As stated, COVID-19 is a systemic disease inducing systemic inflammation and occasionally cytokine storm. This can significantly impact the process of hematopoiesis and hemostasis. During early disease, normal or decreased leukocyte and lymphocyte counts were documented with marked lymphopenia as the diseases progressed, especially in those with cytokine storms and severe disease. In a study of 1,099 patients, lymphopenia, thrombocytopenia, and leukopenia were present in 83.2, 36.2, and 33.7%, respectively, with findings more marked in those with severe diseases (18). Leukocytosis in COVID-19 patients might suggest a bacterial infection or a superinfection with leukocytosis found more commonly in severe cases (11.4%) as compared to mild and moderate cases (4.8%) (18). Similarly, thrombocytopenia has been found to be more common (57.7%) in severe cases in contrast to mild and moderate cases (31.6%) (18). Lymphopenia was also linked with an increased necessity for ICU admission and the risk of ARDS. Thrombocytosis with elevated platelet to lymphocyte ratio may indicate a more marked cytokine storm (57).

Also, coagulation abnormality can manifest in the form of thrombocytopenia, prolonged prothrombin time (PT), low serum fibrinogen level, and raised D-dimer suggesting Disseminated Intravascular Coagulation (DIC) with these changes more marked in those with severe diseases (58). Raised lactate dehydrogenase (LDH) and serum ferritin were also present and correlated with the degree of systemic inflammation. In a study of 426 COVID-19 patients, C-Reactive Protein (CRP) was noted to be increased in 75–93% of patients, more commonly in patients with severe disease. Serum procalcitonin levels might not be altered at admission, but progressive increase in its value can suggest a worsening prognosis. Severe disease is linked to increased ALT, bilirubin, serum urea, creatinine, and lowered serum albumin (59). A study of 1,426 patients showed that Interleukin-6 (IL-6) were raised more in patients with severe COVID-19 than non-severe COVID-19 with progressive rise indicating an increased risk of mortality. Thus, its levels could be regarded as an important prognostic indicator for the extensive inflammation and cytokine storm in COVID-19 patients (60). Other plasma cytokines and chemokines like IL1B, IFNγ, IP10, MCP, etc. have also been found to be elevated in patients with COVID-19 both in severe and non-severe diseases. Additionally, GCSF, IP10, IL2, IL7, IL10, MCP1, MIP1A, and TNFα were increased in patients who require ICU admission which indicates that cytokine storm is associated with a severe disease (61).

### Endocrine and Reproductive

From the available literature there is no doubt that diabetes mellitus is an important risk factor for COVID-19 illness and is associated with increased risk of development of severe disease. Additionally, there are case reports of subacute thyroiditis linked to SARS-CoV-2 infection (62, 63). Based on the statement released from European Society of Endocrinology, patients with primary adrenal failure and congenital adrenal hyperplasia may have theoretically increased susceptibility to infection with higher risk of complications and ultimately mortality but there is no current evidence to support this (64). The dose of steroids may need to be doubled if there is a clinical suspicion of infection in these patients.

Several claims have been made regarding the impact of COVID-19 on male reproductive function, hypothesizing that COVID-19 can cause potential testicular damage either by binding directly to testicular ACE2 receptors, which are highly expressed in the testicles or by damaging the testis indirectly by exciting local immune system (65). A study comparing 81 male COVID-19 patients with 100 age matched healthy adults highlighted the presence of low testosterone levels, high levels of luteinizing hormone (LH), low testosterone/LH ratios, low Follicle stimulating hormone (FSH) to LH ratio, and raised serum prolactin. This may suggest a potential COVID-19 testicular damage affecting the Leydig cells in the testis (66). COVID-19 infected male patients may have reduced sperm count and decreased motility leading to diminished male fertility for 3 months post-infection (67).

## Clinical Presentation in Specific Population

### In Children

A case series of 72,314 cases published by the Chinese Center for Disease Control and Prevention reported that 0.9% of the total patients were between 0 and 9 years of age, and 1.2% of the total patients were between 10 and 19 years of age (68). The most common symptoms found in children are fever, (59%), cough (46%), few cases (12%) of gastrointestinal symptoms, and some cases (26%) showed no specific symptoms initially with patchy consolidation and ground glass opacities in CT chest findings (69). Chilblain-like acral eruptions, purpuric, and erythema multiforme-like lesions have been found to be more common in children and young adult patients mainly with asymptomatic or mild disease (70). Lymphopenia in children is relatively less common which is in direct contrast in cases of SARS in children where lymphopenia was more commonly noted (69).

Multisystem inflammatory syndrome (MIS) is another feared complication of Covid-19 seen in children. Abrams et al. systematically summarized the clinical evidence of 8 studies reporting MIS in 440 children. The median age of patients ranged from 7.3 to 10 years with 59% of all patients being male. The greatest proportion of patients had gastrointestinal symptoms (87%) followed by mucocutaneous symptoms (73%) and cardiovascular symptoms (71%) while fewer patients reported respiratory (47%), neurologic (22%), and musculoskeletal (21%) symptoms. Ferritin and d-dimer were elevated in 50% of patients, and C-reactive protein, interleukin-6, and fibrinogen were elevated in at least 75% of patients. Additionally, 100% of children with cardiovascular involvement reported elevated cardiac-damage markers such as Troponin. Although respiratory manifestation is most frequently expressed in adults, children with MIS exhibited less pulmonary symptoms and more of the other manifestations (71).

### In Pregnant Women

The most common symptoms reported in pregnant women are fever (61.96%), cough (38.04%), malaise (30.49%), myalgia (21.43%), sore throat (12%), and dyspnea (12.05%). Other symptoms found in pregnant women are diarrhea and nasal congestion (72). In a systematic review including 92 patients, 67.4% manifested diseases at presentation with 31.7% having negative RT-PCR though they had features of viral pneumonia. Only one patient required admission to intensive care and 0% mortality. Fetal outcomes were reported as: 63.8% preterm delivery, 61.1% fetal distress, 80% Cesarean section delivery, 76.92% neonatal intensive care admission, 42.8% low birth weight, and 66.67% had lymphopenia (72). There was no evidence of vertical transmission. A study of 41 pregnant women with COVID-19 showed that consolidation was more commonly found in CT of pregnant women in contrast to ground-glass opacities in CT of non-pregnant adults (73). WHO also recommends encouraging lactating mothers with confirmed or suspected COVID-19 to begin or continue breastfeeding including 24-h rooming in, skin to skin contact, and kangaroo mother care especially in immediate postnatal period (74). On July 14th, 2020, Vivanti et al. published the first case of transplacental transmission of COVID-19 from a 23-years-old pregnant woman to her baby (75). Thereafter, more studies reported the possibility of the vertical transmission of COVID-19. In this context, Kotlayer et al. published a systematic review of 38 studies. Out of 936 neonates from COVID-19 mothers, 27 tested positive for the virus indicating a pooled proportion of 3.2% (2.2–4.3) for vertical transmission (7).

### In Immuno-Compromised Population

Due to their impaired immune response, it is not surprising that immunocompromised patients with COVID-19 infection might be at greater risk of developing severe forms of the disease and co-infections in comparison to normal populations. Nevertheless, recent studies showed the association between cytokine storm syndrome and the overreaction of the immune system with COVID-19 raising the possibility that immunodeficient states might alleviate the overexpression of the host immune system and thereby prevent deadly forms of the disease (76). After the RECOVERY trial (77) that showed the efficacy of dexamethasone in lowering the mortality in severe forms of the disease, many questions were raised regarding whether immunocompromised patients have a greater or lower risk of developing severe forms of the disease. In order to address these questions, Minotti et al. recently published a systematic review that included 16 studies with 110 patients presenting mostly with cancer along with transplantation and immunodeficiency. Out of the 110 patients, 72 (65.5%) recovered without being admitted to the intensive care unit while 23 (20.9%) died (76). The authors concluded that immunosuppression in both children and adults seem to have a better disease course in comparison to normal population. One of the limitations of this study is that the conclusion was made only based on qualitative synthesis and no meta-analysis was performed. On the other hand, Gao et al. performed a meta-analysis on 8 relevant studies with 4,007 patients. The study showed that immunosuppression and immunodeficiency were associated with non-statistically significant increased risk of severe COVID-19 disease (78). Additionally, Mirzaei et al. summarized the clinical evidence of 252 HIV positive patients co-infected with COVID-19. The clinical manifestation did not differ from that of the general population. However, out of the 252 patients, 204 (80.9%) were male. Low CD4 count (<200 cells/mm^3^) were reported for 23 of 176 patients (13.1%). COVID-19 symptoms were present in 223 patients with the most common symptoms of fever in 165 (74.0%) patients, cough in 130 (58.3%), headache in 44 (19.7%), arthralgia and myalgia in 33 (14.8%), gastrointestinal symptoms in 29 (13.0%) followed by sore throat in 18 (8.1%) patients (79). The number of deaths accounted for 36 (14.3%). Similar to the general population, immunocompromised, and HIV patients were no different in terms of clinical manifestation or severity. However, the results from these studies should be interpreted with caution and more studies are recommended to establish the link between this particular group of patients with severity of the disease.

## Multisystem Involvement in COVID-19

As evident from the discussion above, SARS-CoV-2 can affect multiple organ systems and produce a wide array of clinical presentation of COVID-19. Certain studies conducted in Europe and United States have shown that COVID-19 can also have a multi-systemic presentation in individuals in form of a multi-system inflammatory syndrome (MIS) which has been found in both children and adults and is known as MIS-C and MIS-A, respectively (80–83).

According to a recent CDC report about MIS-A, it was found that only half of the patients with MIS-A had preceding respiratory symptoms of COVID-19 ~2–5 weeks before (80). The most common clinical signs and symptoms included fever, chest pain, palpitations, diarrhea, abdominal pain, vomiting, skin rash, etc. Nearly all patients had electro-cardiological abnormalities like arrythmias, elevated troponin levels, and electrocardiography evidence of left or right ventricular dysfunction. Even though most patients had minimal respiratory symptoms, chest imaging had features of ground glass opacity and pleural effusion. All patients had signs of elevated laboratory markers of inflammation, coagulation markers, and lymphopenia (80).

MIS-C can clinically mimic Kawasaki Disease (81). By the end of July, about 570 cases of MIS-C with COVID-19 were found in the United States (81). In MIS-C, there is involvement of at least four organ systems, most commonly the gastrointestinal system followed by cardiovascular and dermatological systems (81). Prominent signs and symptoms found in children with MIS-C were abdominal pain, vomiting, skin rash, diarrhea, hypotension, and conjunctival injection. The majority of the children needed ICU admission due to the development of severe complications including cardiac dysfunction, shock, myocarditis, coronary artery aneurysm, and acute kidney injury (81).

## Association Between Clinical Presentations, COVID-19 Severity and Prognosis

Evaluation of 55,924 laboratory confirmed COVID-19 cases in China, the presence of dyspnea, respiratory rate ≥ 30/min, blood saturation levels ≤ 93%, PaO2/FiO2 ratio ≤ 300, lung infiltrates ≥ 50% of the lung fields between 12 and 48 h were associated with severe COVID-19 infection (1). Clinical signs suggestive of respiratory failure, septic shock, or multiple organ dysfunction/failure were associated with critical disease and poor prognosis (1). Individuals at highest risk of severe disease and deaths were patients with age > 80 years and associated co-morbidities such as underlying cardiovascular disease, diabetes, hypertension, chronic respiratory disease, and cancer (1). Another study done with 418 patients in Catalonia (Spain) showed that dyspnea was an important predictor of severe disease while confusion was an important predictor of death, and the presence of cough was strongly associated with good prognosis (84). Advanced age, male sex, and obesity were independent markers of poor prognosis while eosinophilia was a marker of less severe disease (84). The mortality was lower in patients with symptoms of diarrhea, arthromyalgia, headache, and loss of smell and taste sensations while low oxygen saturation, high CRP levels, and higher number of lung quadrants affected on Xray were found to be associated with severe disease and death (84).

## Conclusion

COVID-19 is a viral illness which can cause multi-systemic manifestations. Review of existing literature concludes that SARS-CoV-2 can affect any organ system either directly or indirectly leading to a myriad of clinical presentation. The most commonly affected system is the respiratory system with presenting symptoms of fever, cough, and shortness of breath, etc. Other systems which can be affected in COVID-19 include ENT (sore throat, loss of taste, smell, and sensations, and rhinorrhea), cardiovascular system (chest pain, chest tightness, palpitations, and arrhythmias), gastrointestinal system (anorexia, diarrhea, vomiting, nausea, and abdominal pain), renal (proteinuria, hematuria, and acute kidney injury), neurological (headache, confusion, delirium, and altered mental status), ocular (conjunctival hyperemia, foreign body sensation in the eye, chemosis, and tearing), cutaneous (rash, papules, and urticaria), musculoskeletal system (myalgia and arthralgia), hematological (lymphopenia, thrombocytopenia, leukopenia, elevated inflammatory markers, and elevated coagulation markers), endocrine (low testosterone, low FSH, and high LH) and reproductive system (decreased sperm count and decreased sperm motility). Clinical presentation in specific populations like children, pregnant women, and immunocompromised people may vary which emphasizes the importance of further investigation in order to avoid late diagnosis of COVID-19. Severe multi-systemic involvement in COVID-19 in the form of MIS-C and MIS-A can cause significant morbidity and mortality if undiagnosed. The clinical presentations of respiratory failure, acute kidney injury, septic shock, cardiovascular arrest is associated with severe COVID-19 disease and can result in poor prognosis. In the light of exponentially growing pandemic, every patient presenting to hospital must be tested for SARS-CoV-2 by RT-PCR if resources are available to detect early presentations of diseases even if the features are atypical. Understanding of the various clinical presentations of COVID-19 will help the clinicians in early detection, treatment, and isolation of patients in order to contain the virus and slow down the pandemic.

## Author Contributions

All authors have contributed equally to the work, and all agreed to be accountable for the content of the work.

## Conflict of Interest

The authors declare that the research was conducted in the absence of any commercial or financial relationships that could be construed as a potential conflict of interest.

## Abbreviations

ACC/AHA/HFSA: American College of Cardiology/American Heart Association/Heart Failure Society of America
IL1B: Interleukin 1B
IFNγ: Interferon Gamma
IP10: Interferon-inducible Protein 10
MCP1: Monocyte Chemoattractant Protein 1
GCSF: Granulocyte Colony Stimulating Factor
IL2: Interleukin 2
IL7: Interleukin 7
IL10: Interleukin 10
MIP1A: Macrophage Inflammatory Protein-1 alpha
TNFα: Tumor Necrosis Factor alpha.
